# Long short term memory deep net performance on fused Planet-Scope and Sentinel-2 imagery for detection of agricultural crop

**DOI:** 10.1371/journal.pone.0271897

**Published:** 2023-02-03

**Authors:** Touseef Ur Rehman, Maaz Alam, Nasru Minallah, Waleed Khan, Jaroslav Frnda, Shawal Mushtaq, Muhammad Ajmal

**Affiliations:** 1 National Center for Big Data and Cloud Computing (NCBC), University of Engineering and Technology (UET), Peshawar, Khyber Pakhtoonkhwa (KP), Pakistan; 2 Department of Computer Systems Engineering, University of Engineering and Technology, Peshawar, Khyber Pakhtoonkhwa (KP), Pakistan; 3 Department of Telecommunications, Faculty of Electrical Engineering and Computer Science, VŠB – Technical University of Ostrava, Ostrava, Czechia; 4 Department of Quantitative Methods and Economic Informatics, Faculty of Operation and Economics of Transport and Communication, University of Zilina, Zilina, Slovakia; 5 Department of Agricultural Engineering Peshawar, University of Engineering and Technology, Peshawar, Khyber Pakhtoonkhwa (KP), Pakistan; Forest Research Institute Dehradun, INDIA

## Abstract

In view of the challenges faced by organizations and departments concerned with agricultural capacity observations, we collected In-Situ data consisting of diverse crops (More than 11 consumable vegetation types) in our pilot region of Harichand Charsadda, Khyber Pakhtunkhwa (KP), Pakistan. Our proposed Long Short-Term Memory based Deep Neural network model was trained for land cover land use statistics generation using the acquired ground truth data, for a synergy between Planet-Scope Dove and European Space Agency’s Sentinel-2. Total of 4 bands from both sentinel-2 and planet scope including Red, Green, Near-Infrared (NIR) and Normalised Difference Vegetation Index (NDVI) were used for classification purpose. Using short temporal frame of Sentinel-2 comprising 5 date images, we propose an realistic and implementable procedure for generating accurate crop statistics using remote sensing. Our self collected data-set consists of a total number of 107,899 pixels which was further split into 70% and 30% for training and testing purpose of the model respectively. The collected data is in the shape of field parcels, which has been further split for training, validation and test sets, to avoid spatial auto-correlation. To ensure the quality and accuracy 15% of the training data was left out for validation purpose, and 15% for testing. Prediction was also performed on our trained model and visual analysis of the area from the image showed significant results. Further more a comparison between Sentinel-2 time series is performed separately from the fused Planet-Scope and Sentinel-2 time-series data sets. The results achieved shows a weighted average of 93% for Sentinel-2 time series and 97% for fused Planet-Scope and Sentinel-2 time series.

## 1 Introduction

From the inception of human civilization, agriculture has been regarded as the backbone of social and economic evolution. About 60% of the total population lives in rural areas and is highly dependent upon agricultural activities [1], contributing more than 24% to the Gross Domestic Product (GDP) of Pakistan and commissions half of the labour force. Moreover, it is also the largest source of foreign exchange earnings (https://www.pbs.gov.pk/content/agriculture-statistics). However, the concerned agencies are facing difficulties in accurate crop monitoring and yield estimations [2] due to inaccurate and insufficient data from improper mechanisms. Making the task more challenging is the limited use of technology for generating seasonal crop statistics by the government, resulting in theft, overstocking and illegal trade.

The currently deployed mechanisms in the country are limited to ground surveys and manual measurements, often proving very expensive due to a large number of human surveyors requirements. For policy-level decisions, cultivated land area and yield estimations are essential for determining the amount of food stored or exported to reduce food losses along the food supply chain [3].

Geographic Information System (GIS) has been adopted globally as a decision support system, for a variety of problems. Remote Sensing as a primary component of a GIS is the collection of Earth’s observational data through satellites and airborne sensors. Developed nations have already adopted such systems to surveil their valuable resources [4]. A number of remote sensing satellites are freely available for providing remote sensing data. Sentinel-2, Landsat (launched by United States Geological Survey, NASA), MODIS (Moderate Resolution Imaging Spectroradiometer) are a few of the satellites offering free access to remote sensing data. Each satellite offers unique set of features wiz. spectral, spatial and temporal resolution, number of channels and revisit time. Satellite remote sensing (RS) is considered to be a substantial technique for land cover classification and crop statistics generation [5], over a large geographical scale, providing periodically considerable observations regarding ground objects [6].

The surge of satellite data unlocked countless possibilities for land cover land use statistics and transformation of data into information. The common categorization of remote sensing data includes; Multi-spectral, Hyperspectral and Synthetic Aperture Radar (SAR). Multispectral sensors having a limited number of channels are frequently used for vegetation based studies, due to their simple nature, data availability, and fast processing, as compared to Hyperspectral (Having more than 50 bands) and SAR. Based on multispectral remote sensing, vegetation observations are primarily classified into two main categories [7].

1. The use of spectral information from a single date satellite imagery during the growing season of the crop.
2. Utilization of temporal information from revisiting satellites.

Numerous studies refer to the second approach for achieving satisfactory results in terms of accuracy [8–10], but the challenges and anomalies curbing the use of this technique on regular basis exists. Studies conducted on keeping the temporal features in perspective, use season-wise or annual based generation of crop statistics. The data used in these studies mostly consists of a large number of imagery scenes acquired throughout the year or phonological season of a crop. Based on the overwhelming number of satellite images, the probability of certain anomalies increases over time. Some of these are;

1. The In-situ data used for training a machine learning model needs to be collected each year or season due to uncertainty of weather conditions.
2. Cloudy weather gives rise to the non-visibility of the targeted regions, causing a data gap in acquired temporal data.
3. Machine learning models trained are limited to the year of study and needs to be trained on new training data each year.

The synergy of machine learning and RS over the past two decades resulted in marvellous applications for land cover analysis and crop classification [11–13]. A number of techniques and algorithms have been devised for this purpose. Traditional algorithms include Maximum Likelihood, Support Vector Machines, Minimum Distance and Feed Forward Neural Networks, but the advent of time demands a much more lucrative and accurate modus operandi for the surge of open and commercial satellite data [14]. Our model, Long Short-Term Memory(LSTM) based Deep Neural network was trained for seasonal crop statistics generation using the acquired ground truth data, for a synergy between Planet-Scope Dove and European Space Agency’s Sentinel-2. Five medium resolution Sentinel-2 scenes were adopted in combination with one high-resolution Planet-Scope Dove (Fig 3) [15]. The use of a short temporal frame can be an intuitive and intelligible approach to classify vegetation.

Deep Learning (DL) has emerged as a key tool and most popular approach for many fields, including remote sensing [16]. Artificial Neural Networks is an information processing model, that is inspired by the human brain processing mechanisms and has been waged for many applications [10, 17]. In addition multi-layer deep learning architecture using multi-temporal images is used. LSTM is a special kind of Recurrent Neural Network (RNN) model that is found to be very effective with problems related to sequential data [18–20]. LSTMs are precisely designed for long term dependency problems between the events with time gaps. Their default nature to learn and remember information out-stand them among many other RNNs [21]. They are free from optimization hurdles which is one of the obstacles for simple recurrent neural networks (SRNs) [22]. Problems such as handwriting recognition, language translation, and speech synthesis, analysis of audio, and video data are easily learned and solved by LSTM models [23–25]. The objectives of this study include;

1. Development of an LSTM based model for crop classification in heterogeneous conditions.
2. Validation and verification of the developed algorithm in the field.
3. Design and development of crop classification mechanism using remote sensing through the use of short temporal frame and deep neural networks.
4. Providing a suitable and realistically implementable methodology for crop statistics generation in Pakistan using remote sensing.
5. A comparison between Sentinel-2 time-series and fused Planet-Scope and Sentinel-2 time-series.

## 2 Related work

Constituted by a wide variety of geographic landscape, ranging from fertile plains to deserts and the hard-working populace, makes up a country to be an agricultural state. The main chunk of our economy is agriculture based and is the main source of employment [26]. However, this main sector despite deserving elaborate planning and development has been left unattended and as a consequence, a whole lot of potential is wasted each year. Aslam et al. identified the major cause that the centuries-old methods are still in vogue here leaving the farmers at a disadvantage of low yields and related financial issues [26]. Remote Sensing technology in this regard is a key tool to know the exact yields and other parameters revolving around agriculture and for land cover and land use classification [10] over a large spatial extent.

Yan et al.explained the use of LiDAR technology-one of the powerful tool used for land cover classification in order to carry out better monitoring and surveillance [27]. Keeping the importance of crops in consideration, new research paradigms for the detection, classification and yield estimation are being carried. The yield estimation of one of the major crops, Tobacco was carried out using a Neural network classifier with single date imagery, resulting in an accuracy of 88.49% [28]. Moreover, an accuracy of 99.20% [29] was carried out by using an Artificial Neural Network (ANN) for land cover classification.

The advancements in temporal resolution of satellites, classification and analysis tend to be the new approach for the identification and prediction of various crops. This can be done using the crop phenological cycles [30], provided the In-situ data. Cheng et al. elaborated the utilization of Sentinel-2 and Planet-Scope Dove at a very exquisite level of spatial resolution [31], for the retrieval of varying vegetation phenology stages with respect to different terrains having short-term vegetation seasons. Spatio-temporal remote sensing images incorporated with 3D Convolutional Neural Networks CNN rather than 2D CNN for classification of crops proved to be a novel method in characterizing the different phases and divisions of crop growth [32].

Buscombe et al. represented his research analysis and interpretation of geomorphic processes by implementing the deep convolutional neural networks (DCNNs) demonstrating the general effectiveness of a very fast and existing framework achieving an accuracy level of up to 98% [33]. In addition, Palchowdhuri et al. elaborated the Random Forest Classifier [34] for various crop types via multi-temporal images of world view [35] and Sentinel-2 satellites [36] resulting in 91% of overall accuracy.

C. Pelletier et al. [37] explained their work in a way to prove Temp-CNNs as one of the best for crop types classification via multi temporal satellite imagery. They predicted 13 different classes by the network with an overall accuracy of 93.5%. Pixel based classification model was created taking spectral and temporal features into consideration using Temp CNNs and RNNS. This model utilized temporal inception blocks and fusion of multi satellite imagery to come-up with more richer and abundant features, thus to emanate 98% of accuracy [15]. M. Weiss et al. put forward an empirical approach for the retrieval and postulated that plant traits and agronomical variables can be estimated by remote sensing. In [38], a description of the latest remote sensing techniques specifically for the agricultural sector is provided. Special satellites are designed for agricultural monitoring, including vegetation detection, drought estimation, and statistical collection for crop health. The GeoEye-1, Advanced Land Observation System (ALOS), FORMOSAT-2, Ikonos, QuickBird, GeoFen, SPOT 6 and SPOT 7, and World View are the names of some satellites for agricultural monitoring [27, 39, 40]. Authors in [41] conclude that the use of vegetation indices is helpful in examining specific crop types. The Random Forest (RF) is a non-parametric machine learning algorithm, performing classification with high accuracy results. Moreover, Random Forest algorithm is used in many remote sensing applications with excellent results and high accuracy levels [42–47].

The Deep Learning (DL) is a sub field of Machine Learning (ML) primarily concerned with methods and algorithms inspired by the function and structure of the brain, called Artificial Neural Network also known as ANN. The main advantage of the DL is feature learning, i.e. automatic feature extraction from raw data, along with its features from higher levels of the hierarchy being formed by the composition of lower-level features [48]. The DL has the ability to solve sophisticated problems particularly well and fast, because of the more complex models used, which allow vast parallelization [49]. The highly hierarchical structure and large learning capacity of the DL models result in accurate classification and predictions for a wide variety of complex (from a data analysis perspective) challenges. The techniques of the DL have provided fruitful results in applications ranging from land cover, image classification and speech recognition to anomaly detection [48, 50]. The recent wide applications of deep learning in multiple fields have shown great progress.

Moreover, most of the DL methods use neural network architectures, therefore deep learning models are often referred to as deep neural networks. The term “deep” normally points to the total number of hidden layers in the neural network. Traditional neural networks only contain 2–3 hidden layers, whereas deep networks contain as many as 100 or more.

In 1943, Walter Pitts and Warren McCulloch worked together to create a computer model based on neural networks, same as the human brain. They used a combination of mathematics and algorithms, which were further called **“threshold logic”**.

(https://www.dataversity.net/brief-history-deep-learning/).

Larochelle et al. explained that the DL algorithms are very much helpful in unsupervised data concerning, specifically for the bulk of data. Empirical analysis has shown that data representations obtained from stacking up non-linear feature extractors (as in the DL) offer better ML results, e.g., enhanced classification modelling, a better quality of generated samples by generative probabilistic models [51], and the invariant property of data representation.

The DL has drawn a significant of attention in agriculture. One of its applications in agriculture is crop estimation and image recognition [52], which removes several obstacles limiting fast development in robotic and mechanised agriculture.

## 3 Experimental setup

**Fig 1:**
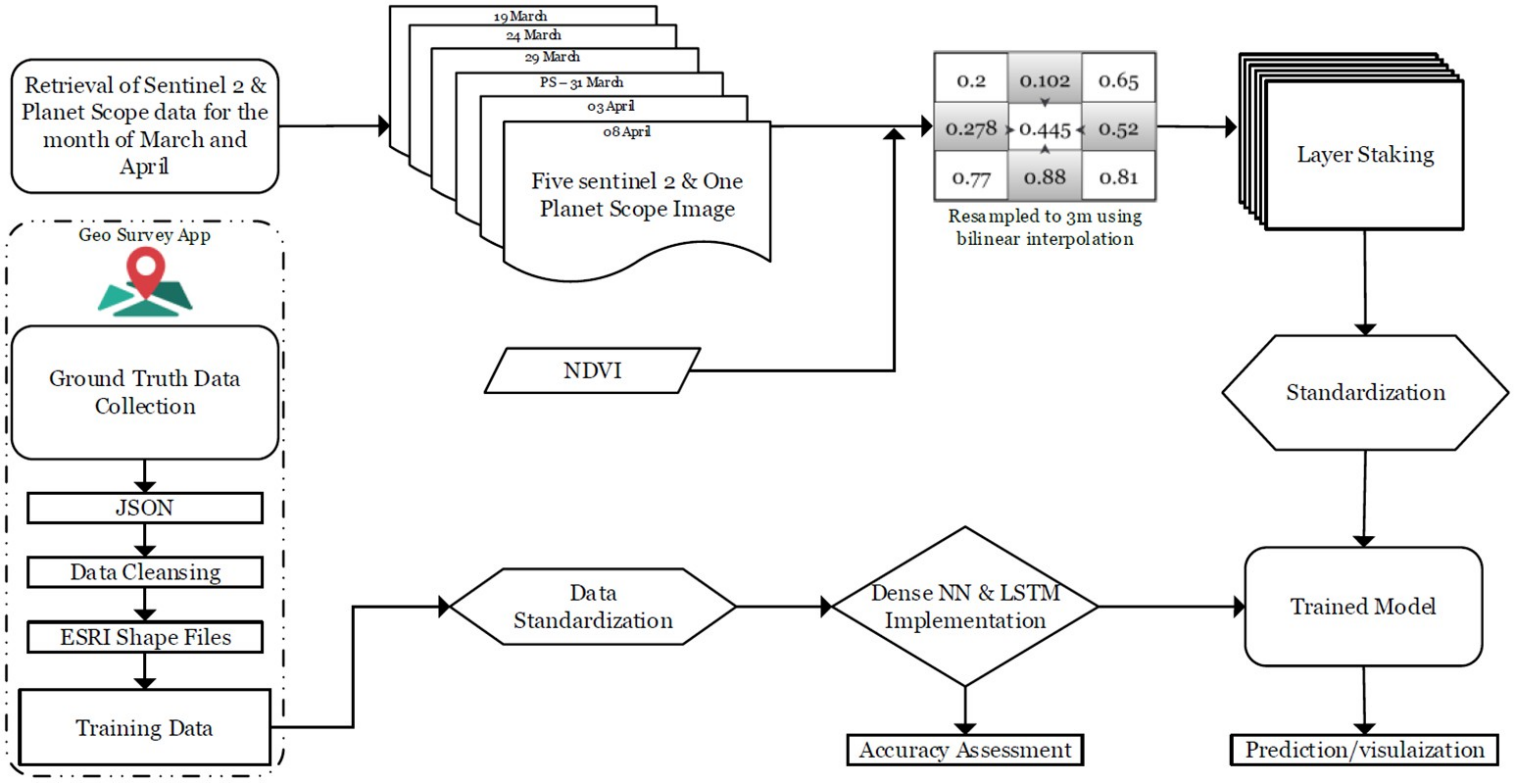
Schematic diagram describing the In-situ data preprocessing and overall data flow.

Fig 1 presents the flowchart, describing the In-situ data preprocessing and overall data flow in a step by step manner. The In-situ data (Training data in the form of field polygons) was collected through our self developed survey application geosurvey, available in google Appstore. After the retrieval of satellite imagery from their respective sources, pre-processing steps such as, resampling, layer stacking and standardization is performed. Training data generated through the collected polygons are fed to our developed LSTM model for training and testing, after which the step of prediction regarding the data-set of satellite images is performed. 2 Separate experiments are performed on the collected data (Table 1). Setup 1 preserves the original spatial resolution of Sentinel-2 collected bands i.e. 10 meters (Fig 3), while a spatial resampling of 3 meters is performed on Sentinel-2 based on Planet-Scope very high resolution (VHR) of 3 meters (Fig 3).

**Table 1:** Experimental setups.

	Data Composition	Spatial Resolution
Setup-1	Fused Planet-Scope and Sentinel-2 Time series	Resampled to 3 meters
Setup-2	Sentinel-2 Time series	10 meters

### 3.1 Area of experimentation

**Fig 2:**
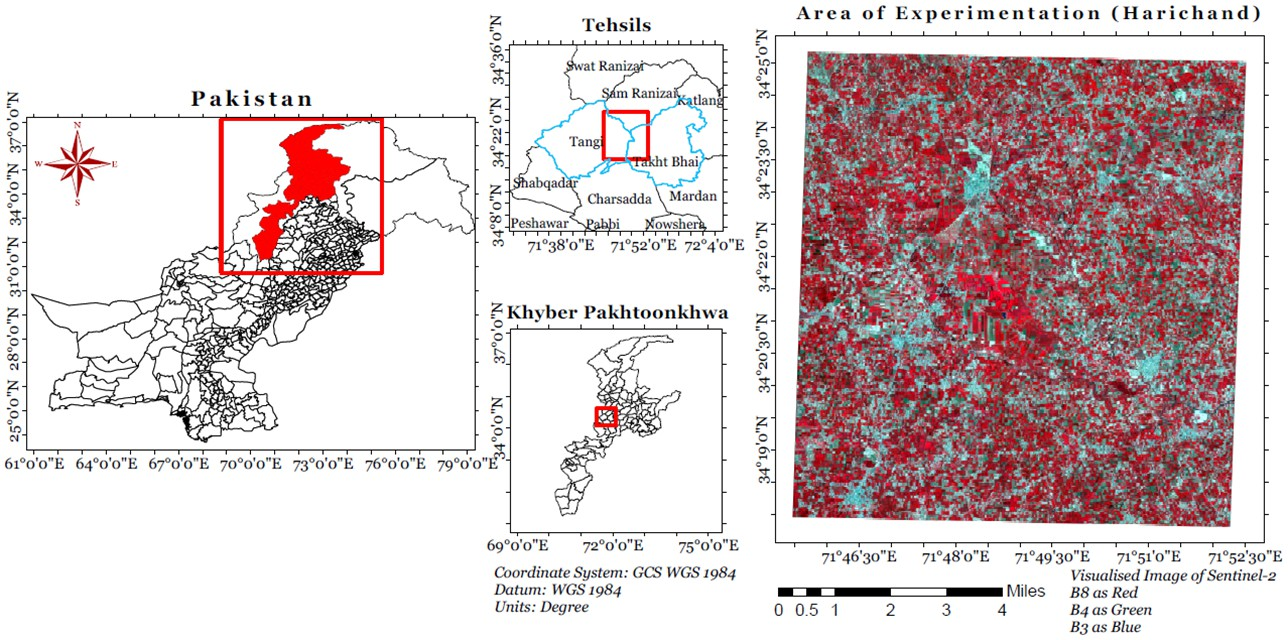
Study area.

In this study, pilot region of Harichand, a town of Charsadda, district Peshawar Khyber Pakhtunkhwa is selected for surveys and data collection shown in Fig 2. This land is known for its diverse vegetation, located at 34°23’2N, 71°48’18E and has an altitude of 381 metres (1253 feet). The surface of Harichand is mostly plain and suitable for agriculture, playing a vital role in the consumable vegetation production in the province and the livelihood of the local farmers. The soil is mostly loamy and fit for vegetation.

The wide range of vegetation includes Mustard, Onion, Tomato, Eggplant, lychees, Red beans, Honey melon, Pumpkins, Peach, Sugarcane, Maize, Ridged gourd, Apple gourd and Loquats/Japanese plum. In addition, some other classes are also observed, like buildings, Water canals and roads.

### 3.2 Data collection

**Fig 3:**
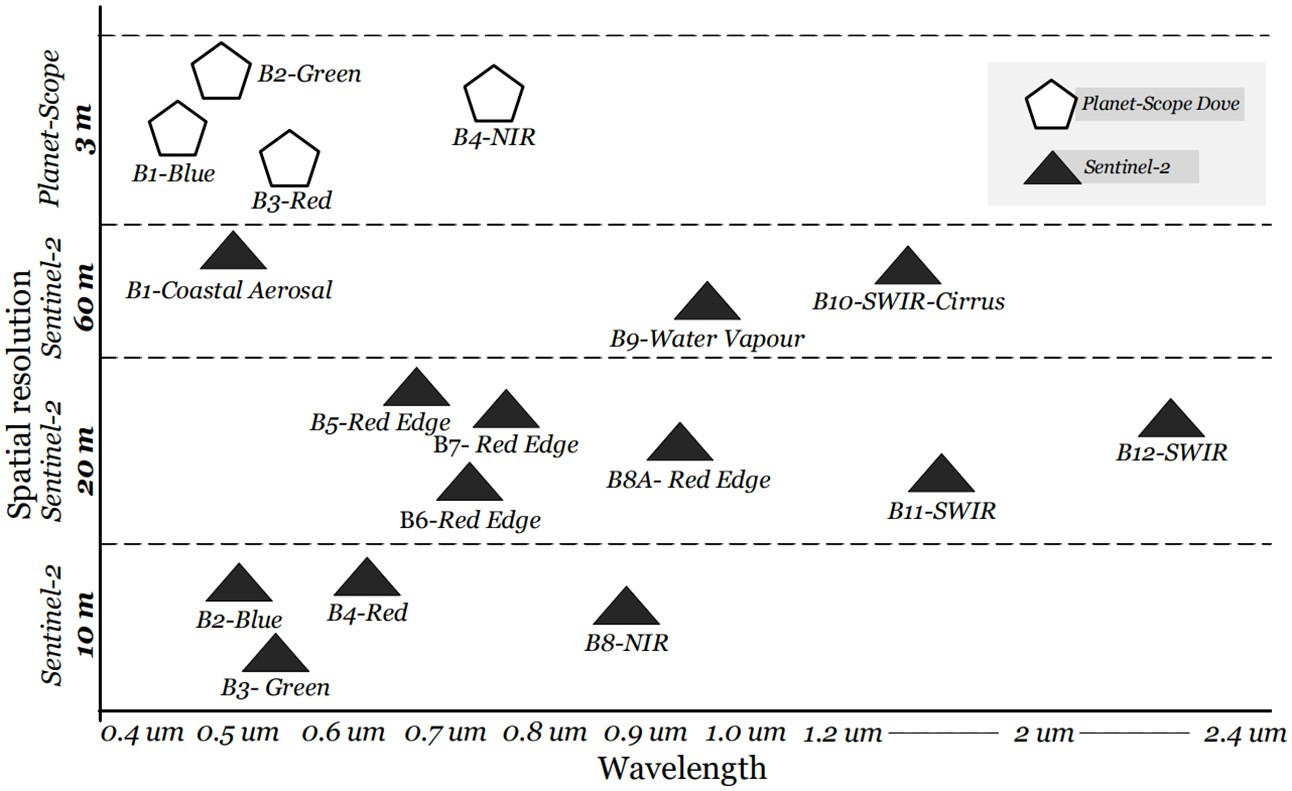
Sentinel-2 and Planet-Scope bands distribution according to EM spectrum and spatial resolution.

**Fig 4:**
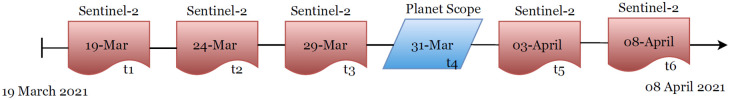
Five Sentinel-2 and one Planet-Scope scenes acquired over the area of interest, Harichand Charsadda. The imageries have been stacked in temporal arrangement for LSTM.

Sentinel-2 imagery is used along with planet scope due to the latter’s high resolution of 3-meters. Moreover, the former’s Multi-Spectral Instrument (MSI) constitutes thirteen spectral bands with three different spatial resolutions (Figs 3 and 4).

**Fig 5:**
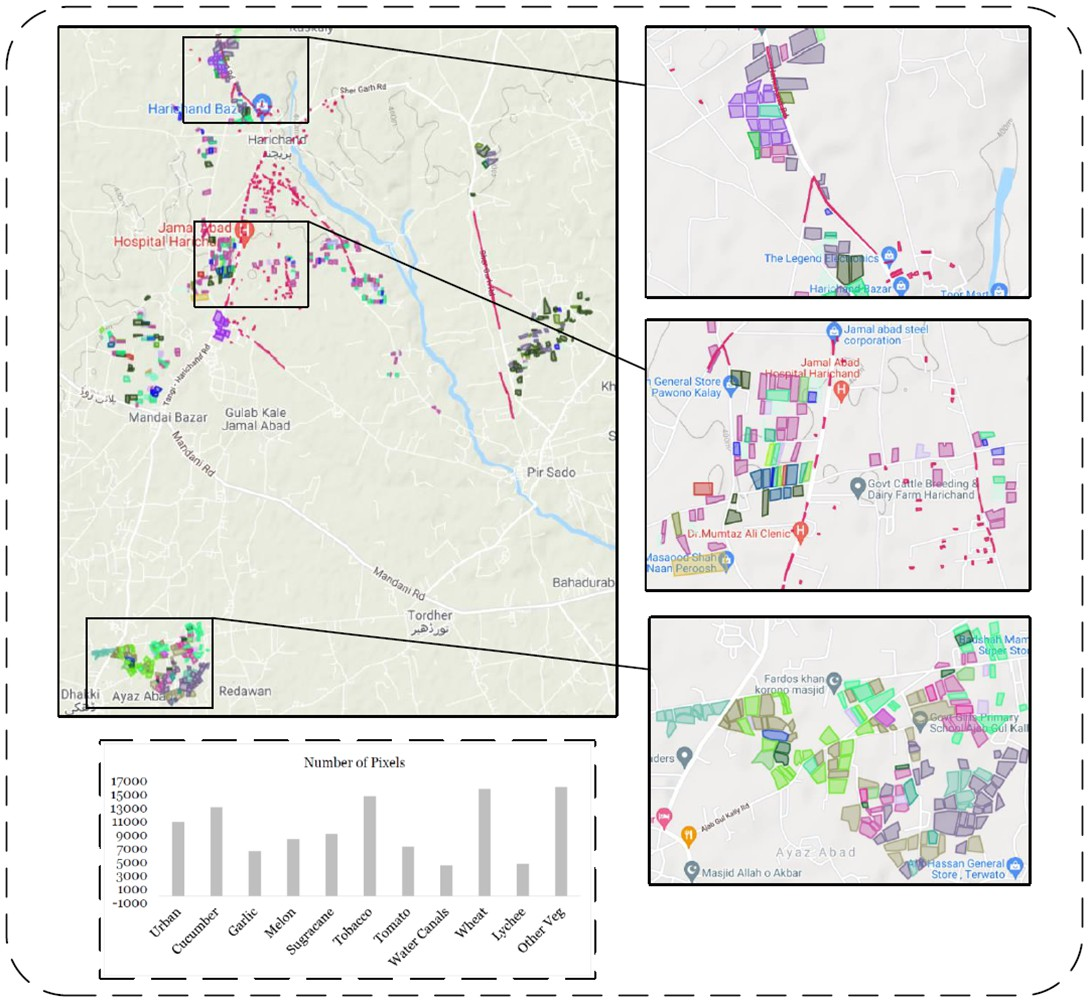
Collected ground truth data (ESRI shapefiles and number of pixels collected during survey).

The retrieved ground truth data (GTD) comprise 11 different classes and their related data is archived from the selected region. Since the area has diverse vegetation, therefore, data for intermix vegetation is also observed. Images of all classes are obtained and the sum of all classes pixels are 107,899. Each class with their pixels information are given in Fig 5.

Survey is conducted in the start of 2021 from January to the mid of April. The distribution of fields in the area are erratic and the phonological cycle of all these vegetables are different and are planted randomly. All crops reach their mature stage in the middle of April and cultivation starts at the end of April. All fields are cultivated randomly.

#### 3.2.1 Data pre-processing

Data pre-processing appears to be an important phase for the purpose of upcoming experiments, therefore they are done under different phases so that everything is aligned for the future framework. The data pre-processing include two main section and are discussed as following;

#### 3.2.2 Pre-processing on In-situ data

Ground truth data that was collected while conducting surveys as discussed earlier, need to be preprocessed before feeding the data to train machine learning models. Following preprocessing, steps were involved in order to get maximum error free data.

Polygons collected during the ground survey were shrunk from the edges using Google Firebase [53], a real-time database developed specifically for the analysis of the ground surveys. The main idea behind this is to avoid outliers from the collected classes. Furthermore, polygons were visually reanalyzed to check for human errors such as overlapping polygons of the same regions and incorrect geometry of polygons.

Polygons were drawn over the satellite image that was retrieved, Further, they were observed by the reflectance of different bands combinations such as: combination of band4(red), band3(green) and band2(blue) of sentinel-2 satellite image gives an RGB(nature colour) image. More often, it is used to monitor the health of crops and is particularly good at highlighting dense vegetation that appears as dark green [54]. This helps in refining our ground truth collected data.

#### 3.2.3 Pre-processing on satellite imagery

In our experimental setup, only 3 out of 13 bands have been used keeping in view the availability of channels in Planet-Scope Dove. These chosen bands comprises of Red, Green, Near-Infrared (NIR) and Normalized Difference Vegetation Index (NDVI).

Satellite imagery is retrieved under different levels, each level requires necessary corrections before further processing to be performed. Our retrieved satellite imagery is of level-2A and ‘Surface Reflectance’ in the case of Planet-Scope Dove, already been corrected for radiometric and atmospheric corrections [28]. Sentinel-2 and Planet scope both comprises different bands with different spatial resolutions ranging from 10–60 meters and 3 meters respectively. For the purpose of layer stacking, sentinel-2 imagery was resampled to the spatial resolution of 3 meters so that all the data have the same pixel size of 3 meters. Bi-linear interpolation was used in Spatial re-sampling which is considered to be the state of art in improving the quality of image without disturbing the image information [55]. Bi-linear interpolation takes a mean of 4 adjacent pixels and calculate the interpolated value and further assigns it to the unknown pixel.

Vegetation index such as NDVI is a measure of the health of a plant, based on how the plant reflects light at certain frequencies [55]. The NDVI is calculated for both sentinel-2 and planet scope imagery the following way;

$$NDVI=\frac{\left(NIR-RED\right)}{\left(NIR+RED\right)}$$

With the help of temporally stacked imagery, reflectance from each class can be recorded on different stages of plants growth. This approach not only improves the classification of the area but also help in increasing the number of features for model training.

#### 3.2.4 Data set

The data collected from the survey of the pilot region shown in Fig 5 was divided into Three subsets, namely training, validation and testing sets. They have an overall 70% for training 15% for validation and 15% for testing of the sample dataset. Moreover, the dataset consists of multispectral imagery from two different satellites i.e Sentinel-2 and Planet-Scope. The train, validation and test split was performed manually by sperating training polygons from validation and test polygons. This was done to ensure Spatial Un-mixing and avoid spatial auto-correlation during training of the model.

The Sentinel-2 provides 13 different spectral bands with a temporal resolution of 5 days, whereas the Planet-Scope can provide 4 different spectral bands with a temporal resolution of 1-day. For our experimental setup, three bands including green, Red and Near Infra-Red with 3 m and 10 m spatial resolution were selected for Planet-Scope and Sentinel-2, respectively. The total number of classes and pixels, collected through ground surveys are presented in Fig 5.

### 3.3 Model development

The LSTM Network is an advanced RNN and a sequential network, that allows information to persist. The LSTMs were introduced by Hochreiter and Schmidhuber (1997) and were further refined and popularized by many people in the following work. They worked enormously well on a large variety of problems, and are now widely used. The LSTM has feedback connections and is capable of handling the vanishing gradient problem faced by RNNs. It can operate on single data points (such as images), and an entire sequence of data (such as speech or video) as well.

**Fig 6:**
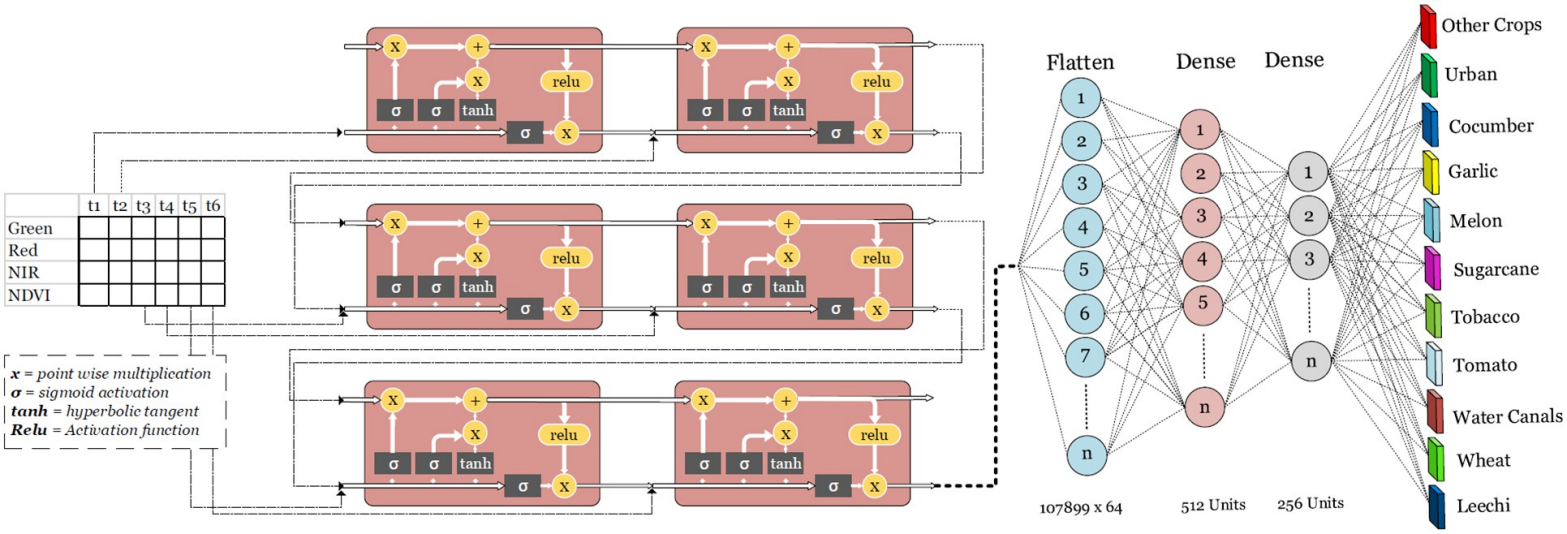
Long short term memory deep neural network.

For example, the LSTM is applicable to tasks such as; unsegmented, connected handwriting recognition, speech recognition and anomaly detection in network traffic or IDSs (intrusion detection systems). The LSTMs are also used to capture Long-term temporal dependencies in a much effective way, without suffering from many optimization hurdles. We used the LSTM for the purpose of classification and model training followed by a fully connected neural network shown in Fig 6 below. A single layer of the LSTM with total of 512 units. Each LSTM unit is composed of the cell, an input gate output and a forget gate [18]. A flatten layer is used to flatten the output of the LSTM which is then fed into a fully connected two layers architecture with hidden units of 256 and 128 respectively. With each hidden layer, an extra layer of batch normalization is used to standardize each output from the hidden layer [56], besides this, a drop out of 0.2 is added for regularization. Each hidden layer is activated by a rectified linear unit (ReLU) function which is considered to be state of the art for Neural networks [57]. Total number of trainable parameters are calculated to be 1,225,995.

Our model is trained with a batch size of 32 and total epochs are set to 25. In order to prevent the model from over-fitting, early stopping is used with patience 5. Initially, the default learning rate of 0.01 with a decay of 0.1 on every 5 epochs was given to the model. Reduce learning rate on plateau function keeps an eye on the learning rate and it gradually decreases the learning rate with time so that it does not overshoot at any instant. The Adam optimizer was observed to give the best results between Sarcastic Gradient Descent (SGD) and RMSprop.

## 4 Validation criteria

Since this is a tricky part and needs a thorough understanding of the data. Relying on only overall accuracy for the credibility of the classifier is not enough therefore different parameters were judged for the purpose of validation which is described below;

### 4.1 Precision

Precision explains the fidelity of the classifier, as it is calculated by taking ratio between true positive to the sum of the true positive and false positive.

$$Precision=\frac{\text{True}\;\text{Positive}}{\text{True}\;\text{Positive}\;\text{+}\;\text{False}\;\text{Positive}\;}$$

### 4.2 Recall

Recall basically provides information regarding the classifier’s perfection. It is defined as the ratio of true positives to the sum of true positives and false negatives for each class.

$$Recall=\frac{\text{True}\;\text{Positive}}{\text{True}\;\text{Positive}\;\text{+}\;\text{False}\;\text{Negitive}\;}$$

### 4.3 F1-score

It is the weight harmonic mean of precision and recall ranging from 1.0 to 0.0 where 1.0 is a good F1 score and 0.0 is worst case.

$$F1Score=2*\frac{\left(\text{Recall}\;*\;\text{Precision}\right)}{\left(\text{Recall}\;\text{+}\;\text{Precision}\;\right)}$$

### 4.4 Overall-Accuracy

It is the ratio of sum of all correctly classified training data pixels to the total number of training data pixels.

$$OverallAccuracy=\frac{\left(\text{Number}\;\text{of}\;\text{all}\;\text{correctly}\;\text{classified}\;\text{pixel}\right)}{\left(\text{Total}\;\text{number}\;\text{of}\;\text{Pixels}\;\right)}*100$$

## 5 System specification

For the purpose of model training a virtual server cloud in amazon web service (AWS) is used called instances. Following are the AWS instance specifications.

- Service Name: General Purpose GPU Extra Large
- API Name: P2.XLarge
- GPU Memory: 12 GB
- GPU Model: NVIDIA Tesla K80 Accelerator
- Memory: 64 GB
- Virtual CPU: 4
- Instance Storage: Amazon Elastic Block Store (EBS)
- Network Performance: High

## 6 Results and discussion

Our model was trained in a rigorous manner keeping an eye on overall hyperparameters so, that models won’t get overfit or underfit for getting perspicacious results. Methods such as early stopping can be very effective in this regard and with the help of validation overfitting can be observed. If the model converges, training is stopped by using early stopping callbacks [58]. Leslie N. Smith et al. [59] elaborated that learning rates play a vital role in tuning Deep neural networks and is considered to be one of the most important hyper-parameters. Therefore special care of training iteration is taken and as a result, the Reduce learning on plateau function was introduced in the same work. C. Pelletier et al. [37] elaborated their work in a way to prove Temp-CNNs as one of the best for crop types classification via multi temporal satellite imagery. They predicted 13 different classes by the network with an overall accuracy of 93.5%.

Pixel based classification model was created taking spectral and temporal features into consideration using Temp CNNs and RNNS. Our proposed model resulted in an accuracy of 97% for 11 classes as compared to C. Pelletier et al’s 93.5% for 13 classes, using Fused Planet Scope and Sentinel-2 (Setup-I) with re-sampled 3 meter resolution and 92.97% for Sentinel-2 only time series with 10 meter resolution.

### 6.1 Fused Planet Scope and Sentinel-2 (Setup-I)

**Fig 7:**
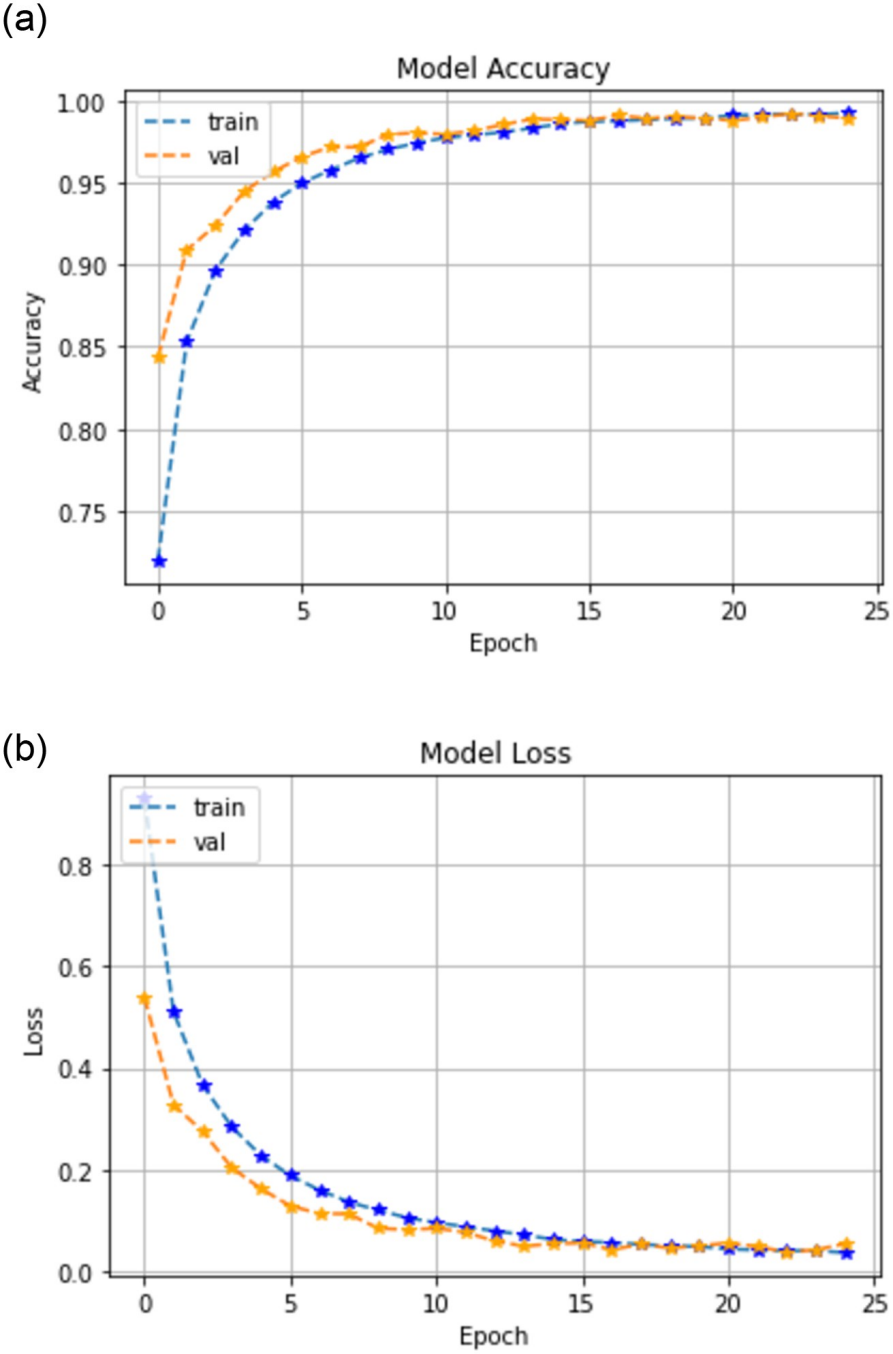
Model accuracy and model loss for fused planet-scope and Sentinel-2 data.

With the trained model and hyper-parameters, an overall accuracy of 97.00% was achieved with a loss of 0.058% on the test data. The accuracy and loss of the model are shown in Fig 7. Fig 7 shows that the training (Blue) and validation accuracy (Orange) increases with the increment in epochs. At 25th epoch the model results in maximum accuracy of 97.00%. Reciprocally the loss decreases with the increasing epochs and at 25th epoch a model loss decreases to 0.058%. Results generated were in the form of a classification report elaborated in Table 2. In this table it can be clearly observed that the proposed model resulted in plausible classification performance. Precision and Recall for class cucumber and tomato were recorded to be 99%, whereas for Wheat, Melons, Water Canals and other Vegetables were recorded as 98% & the recall observed for these classes was upto 99%.

**Table 2:** Classification report Planet scope+S2.

	Precision	recall	f1-score	support
Cucumber	0.96	0.98	0.97	3929
Garlic	0.99	0.90	0.95	1946
Melon	0.95	0.98	0.96	2544
Lychee	0.98	0.98	0.98	1431
Other Vegetation	0.96	0.97	0.98	4862
Sugarcane	0.99	0.98	0.96	2725
Tobacco	0.99	0.99	0.99	4319
Tomato	0.98	0.94	0.99	2174
Urban	0.99	0.98	0.96	3279
Water Canals	0.95	0.98	0.98	332
Wheat	0.96	0.99	0.97	4829
**Accuracy**			0.97	32370
**Macro Avg**	0.97	0.97	0.97	32370
**Weighted Avg**	0.97	0.97	0.97	32370

### 6.2 Only Sentinel-2 results

**Fig 8:**
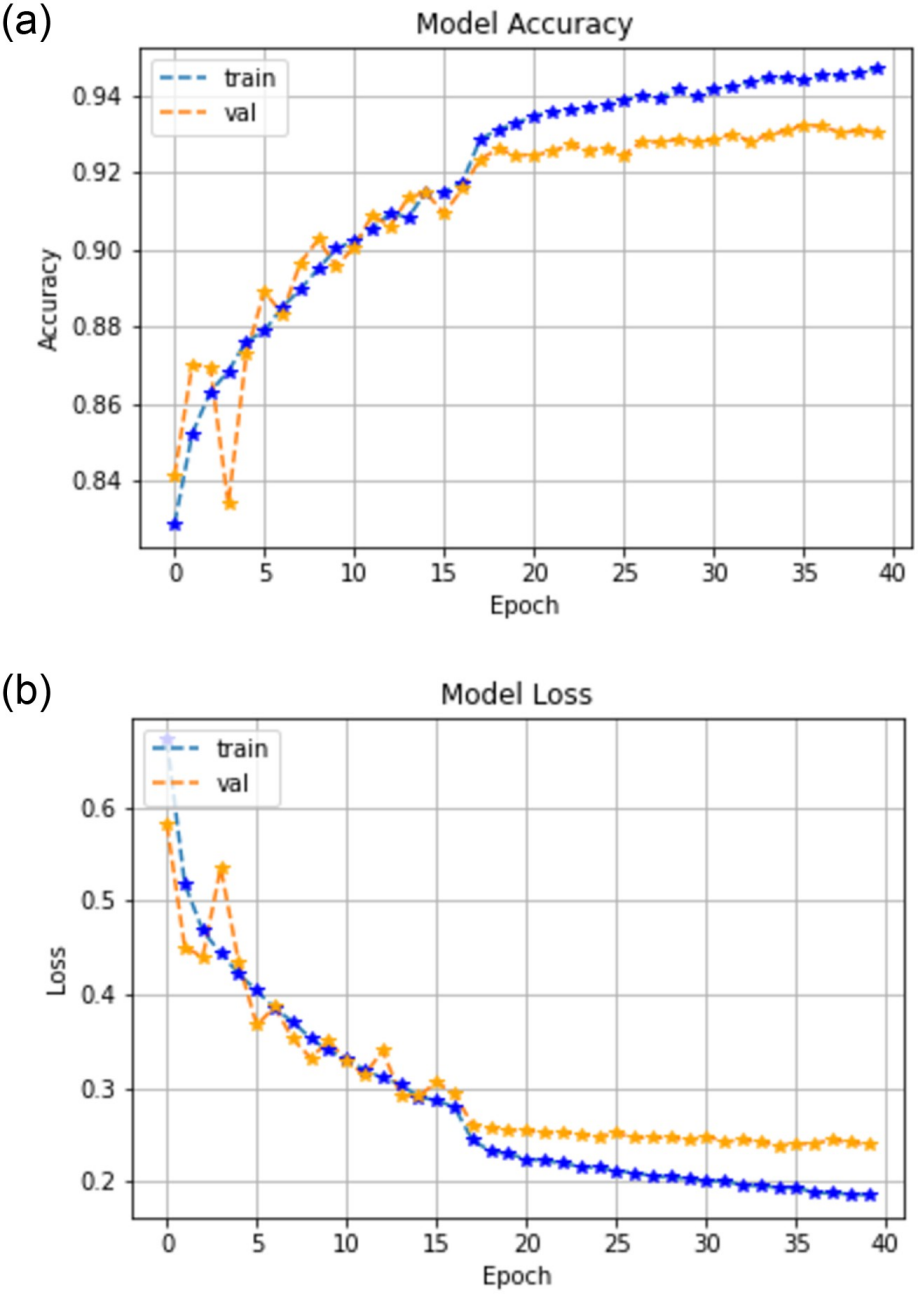
Model accuracy and model loss.

Without the fused Planet Scope imagery, sentinel 2 timeseries achieved an overall accuracy of 93.0% with a loss of 0.23%. Model accuracy and loss are shown in Fig 8. As can be seen in Table 3, with the test accuracy of 93.0% the model under performs for Sentinel-2 time series data. This may be due to small field sizes (less than 0.2 ha) in the region of interest, because fo which there is a lot of spatial mixing between the samples. Fig 8 shows model accuracy and model lose, where it can be seen that around 17th epoch an accuracy of 93.0% is with a loss of 0.23%.

**Table 3:** Classification report only Sentinel 2.

	Precision	recall	f1-score	support
Cucumber	0.83	0.85	0.84	1061
Garlic	0.92	0.83	0.87	177
Melon	0.76	0.72	0.74	262
Lychee	0.89	0.76	0.82	315
Other Vegetation	0.91	0.92	0.91	2866
Sugarcane	0.83	0.64	0.72	219
Tobacco	0.92	0.94	0.93	362
Tomato	0.98	0.98	0.98	297
Urban	0.91	0.95	0.93	4098
Water Canals	0.77	0.49	0.6	215
Wheat	1.00	1.00	1.00	4531
**Accuracy**			0.93	14403
**Macro Avg**	0.88	0.83	0.85	14403
**Weighted Avg**	0.93	0.93	0.93	14403

### 6.3 Impact of spectral indices

Neural networks are series of data-hungry algorithms that try to mimics dense neural operations performed by the central nervous system of the human body. It can observe minute changes and anomalies while making relationships in the bulk of data provided. Initial layers of the neural network start working by learning about simpler and clear features whereas the deep layers learn about more hidden patterns and complex features. In remote sensing different spectral indices are calculated such as the NDVI for the detection of green vegetation and are fed as an extra feature to deep neural networks stacked along with multi spectral imagery.

### 6.4 Visual analysis

**Fig 9:**
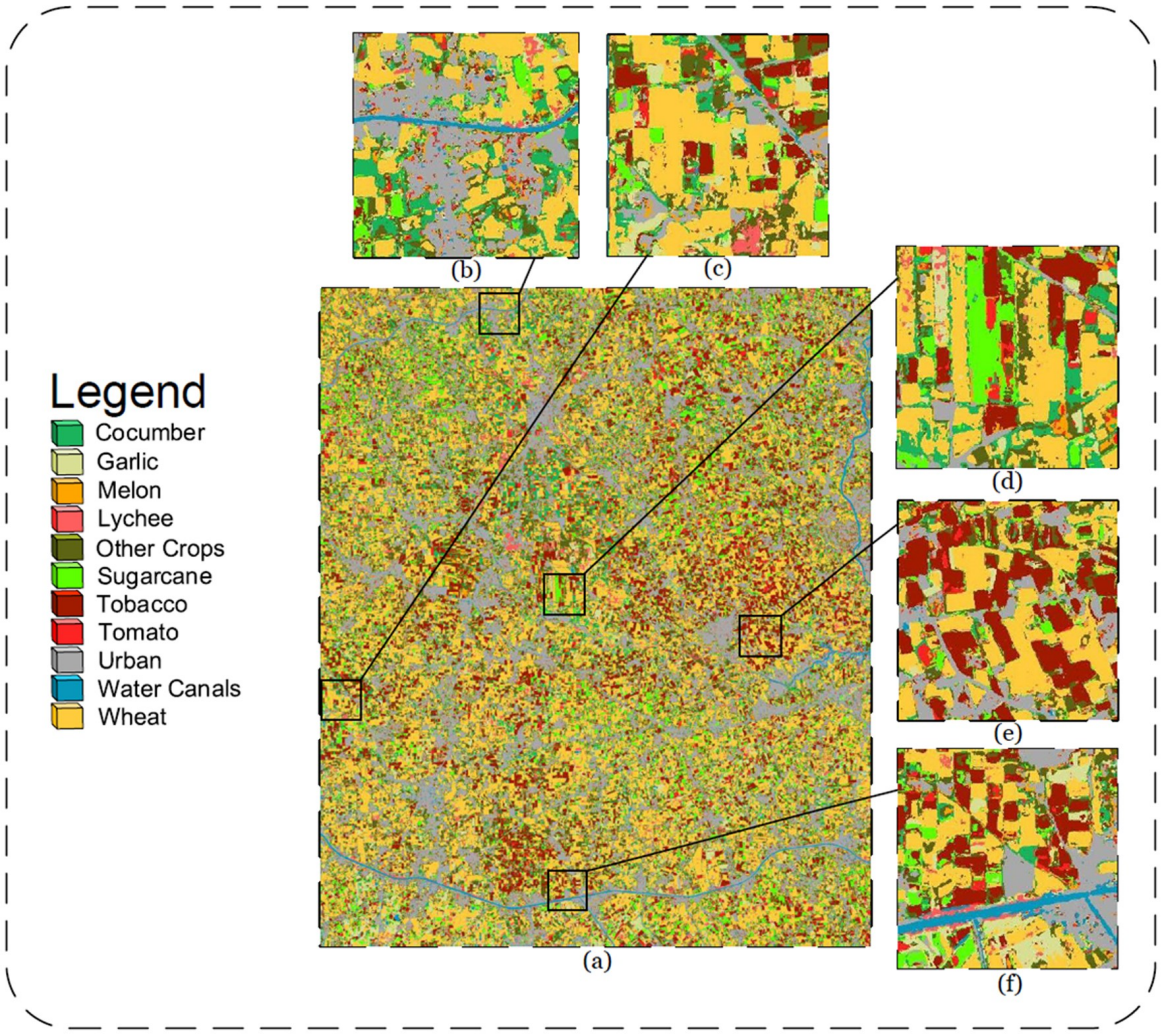
(a) Classified Map of Harichand area showing results for 11 distinct Classes. (b) (c) (d) (e) and (f) Enlarged perspective of the classified map.

Fig 9 shows the predicted map using the trained LSTM model for Setup-1. The visual results of overall region of interest is presented in Fig 9(a), while Fig 9(b) manifests magnified location in the region showing a water canal, field of cucumber, urban areas, wheat and sugarcane. Similarly Fig 9(c), 9(d) and 9(e) maps fields of tobacco, sugarcane, orchards of lychees, wheat, urban areas and cucumber. An intermix between the fields of garlic and wheat can be observed in Fig 9(f), due to the fact of mixed vegetation fields in the area.

## 7 Conclusion

In this work we presented our Long Short-Term Memory based Deep Neural Networks, for crops classification. For this purpose an area of interest was chosen and surveys were conducted for In-situ data collection. A comparison between fused planet-scope and sentinel-2 time-series (Setup-1) and Sentinel-2 only time series (Setup-2) was performed. The proposed model for Setup-1 give an overall test accuracy of 97% for 11 distinct classes, including Urban areas and Water canals. We further analyzed the visual aspects of the predicted satellite data, and found the results to be distinctive in nature. The work also explores the advantage of fused super-resolution Planet-Scope and high resolution Sentinel-2 data for crop classification. The clear distinction in categories is an indication of the extraordinary performance of the LSTM (a type of Recurrent Neural network) deep neural networks for the fusion of Planet-Scope and Sentinel-2 data in a short temporal frame. The LSTM based networks requires temporal data, for which a short temporal frame was selected in the region of experimentation, based on the collective phonological cycle of vegetation in the vicinity. This approach makes our methodology up for a realistic implementation in a large geographical area. This study presents the importance of resampling Sentinel-2 to super-resolution Planet-scope Dove, approaching an improvement of 11.11% in spatial resolution and its relationship with field boundaries. This study entailed the pixel based classification in the region under observation, however the lucrative results in object based classification can be achieved using the LSTM based Deep Neural Networks in the future.

## Supporting information

S1 File (BST)
